# Understanding the Environmentally Sustainable Behavior of Chinese University Students as Tourists: An Integrative Framework

**DOI:** 10.3390/ijerph20043317

**Published:** 2023-02-14

**Authors:** Zhihui Wang, Liangzhen Nie, Eila Jeronen, Lihua Xu, Meiai Chen

**Affiliations:** 1 School of Landscape Architecture, Zhejiang A & F University, Hangzhou 311300, China; 2 Faculty of Education and Psychology, University of Oulu, FI-90014 Oulu, Finland

**Keywords:** environmental awareness, value-belief-norm theory, environmentally sustainable behavior, university students, environmental values

## Abstract

The purpose of this study is to develop a theoretical framework by integrating the value-belief-norm (VBN) theory with environmental awareness in measuring Chinese university students’ environmentally sustainable behavior toward tourism destinations. University students tend to engage in sustainability efforts since their values and beliefs are still being formed. The participants were 301 university students from a university in eastern China. The empirical findings demonstrate that: (1) environmental awareness has positive influences on biospheric value, altruistic value and egoistic value; (2) biospheric value positively predicts the new ecological paradigm (NEP), whereas altruistic and egoistic values do not; (3) the NEP, awareness of consequence and personal norms play an important mediating role. Results indicate that extended VBN can explain students’ environmentally sustainable behavior. This research supports the growth of sustainable tourism and has a number of practical implications for universities and the relevant environmental departments to promote university students’ involvement in sustainable tourism.

## 1. Introduction

As China enters the 21st century, its tourism industry is growing rapidly. According to the Chinese Ministry of Culture and Tourism (2019), the tourism industry’s total contribution to China’s gross domestic product (GDP) in 2018 was 9.94 trillion Chinese yuan renminbi (CNY), accounting for 11.04% of the GDP [1]. Tourism has become one of the pillar industries of China. However, the tourism industry has been negatively impacted by COVID-19 since it began. During the post-COVID era, travel has declined substantially, yet people’s desire to travel has increased because of restrictions imposed on traveling. In the short term, the pandemic caused the tourism industry to concentrate on domestic tourism, while in the long run, it must emphasize safety and sustainability to balance its environmental impact [2]. The idea of sustainable tourism development was first promoted by the World Tourism Organization (WTO) in 1993. During the World Conference on Sustainable Tourism Development in Spain in 1995, the United Nations Educational, Scientific and Cultural Organization (UNESCO), the United Nations Environment Programme (UNEP) and the World Trade Organization (WTO) adopted the Charter for Sustainable Tourism Development, as well as the Action Plan for Sustainable Tourism Development, establishing the sustainable development model as the dominant model for tourism [3,4]. The notion of sustainable tourism has been included in the tourism agendas of many nations [5]. In a post-pandemic setting, a sustainable tourism strategy may be the new normal [6].

Tourism improves people’s living standards, brings economic benefits and promotes employment [7,8,9,10], but it also has harmful effects on the environment. Irreversible and destructive effects on the ecological environment are weakening both local people’s living environment and the tourism environment worldwide. Changing human behavior ultimately makes sustainable development possible since human action is at the root of environmental problems. The promotion of environmentally sustainable behavior among tourists becomes increasingly important as environmentally damaging tourism activities increase [11,12]. Researchers have found that individuals’ negative environmental behaviors may be caused by a lack of environmental awareness [13,14,15]. Environmental awareness, which enables people to comprehend how their decisions impact the environment, is recognized as the primary means to prepare them for environmental issues [16]. According to Van der Werff et al. [17], motivation for eco-friendly behavior can be interpreted as intrinsic motivation based on commitment; they compare this type of motivation to a personal norm. Those with a positive attitude toward nature and a close connection to the environment also strive to protect it. High environmental awareness facilitates sustainable tourism [18]. Thus, analysis of how environmental awareness affects travelers’ environmentally sustainable behavior is crucial. At an individual level, environmentally sustainable behavior includes acts that are concerned with the environment, such as utilizing environmentally friendly items, making green purchases and encouraging others to do the same [19].

Many studies have investigated the environmental behavior of individuals, such as the responsible behavior of tourists towards a destination [20,21,22] and; the environmental friendliness of locals and other tourists [23]. Several theoretical frameworks were applied in these studies, such as the theory of reasoned action [24], the theory of planned behavior [25], the VBN theory [26] and the norm activation model [27]. Two main motivational strategies can be used to sum up behavioral research in favor of sustainable development. The first is a pro-social motivation that draws from Schwartz’s norm activation theory [27,28] and value system and was further developed by Stern’s Value-Belief-Norm Theory [29]; the second is a self-interest-focused theory that primarily draws from Arjan’s Theory of Planned Behavior [25]. The New Ecological Paradigm [30], a widely used environmental attitude scale, is one of the most well-known environmental concern theories. Stern et al. [31] later developed the Norm Activation Theory (NAT) to incorporate the New Ecological Paradigm (NEP). This theory, which Stern et al. [31] named Value-Belief-Norm (VBN) Theory, contends that behavior is predicted by personal norms based on values-formed beliefs. The VBN theory is frequently employed to examine how individual values relate to beliefs, norms and behavior toward the environment [26,29]. In general, these studies have aided in understanding tourism behavior in nature [32,33], but there is very little research information on young people’s views on environmentally sustainable tourism-related behavior.

China’s domestic tourism recovery has been largely driven by younger travelers after the epidemic. Even though they are young and have limited finances, Generation Z is targeted by tourism [34]. “Post-95s” and “post-00s” are defined as those born between 1995–2009 in China, corresponding to western “Generation Z”. The Z population of China currently numbers 149 million. According to experts, this sizable group may provide 40% of the nation’s total power consumption [35]. Tourism businesses are still expecting restrictions to be eased even though the COVID-19 pandemic has not ended yet. High aspirations are placed on Generation Z, particularly by big travel organizations, such as online travel firms. University students who are part of Generation Z are seen as a creative force whose decisions may lead to the development of novel attitudes [36,37] and influence a new general tourist strategy [38]. As an important part of the tourism market, university students’ attention to tourism and the environment plays an important role in the sustainable development of tourism destinations in the future [39]. They carry the weight of environmental problems caused by past and current indifference to nature and the environment. Considering that university students’ values and beliefs are under transition, they are an excellent population for testing and expanding the VBN theory [40]. As a result, this research limits its sample to university students and aims to answer two main questions:

**Q1.**  *How does environmental awareness affect university students’ environmental values?*

**Q2.**  *How do environmental values, beliefs and personal norms influence university students’ environmentally sustainable behavior toward tourism destinations?*

The goal of the study is to develop a theoretical framework by integrating the VBN theory [26] with environmental awareness in measuring Chinese university students’ environmentally sustainable behavior toward tourism destinations. The purpose is to support the planning of young people’s environmental education and environmental political decisions by providing a thorough understanding of what makes university students commit to environmentally sustainable behavior. To accomplish this purpose, data was collected via survey questionnaires and the correlations between variables were explored via structural equation modeling analysis.

## 2. Literature Review and Hypotheses

### 2.1. Environmental Awareness

The concept of environmental awareness was developed in the late 1960s [41]. It implies comprehension of how people and the environment interact [42]. It develops in the context of society based on reflection of experiences, emotions, ideas, beliefs and knowledge [42], and affects people’s experiences and behavior [43]. Recently, environmental awareness has attracted attention for its beneficial effects on tourists’ environmentally sustainable behavior [44,45,46]. Studies show that those with high levels of environmental awareness tend to adopt responsible environmental behaviors [18,47,48]. As the importance of environmental awareness for environmental behavior becomes more widely understood, the understanding of the interaction between the environment and tourists and the effects of the interaction on tourists’ environmental behavior has not increased to the same extent.

As Kellstedt et al. [49] found, compared to the elder generation, younger people are more attentive to environmental issues. Thus, as the most educated of potential tourists, university students can make an important contribution to environmental protection related to sustainable tourism.

### 2.2. Value-Belief-Norm (VBN) Theory

**Figure 1:**
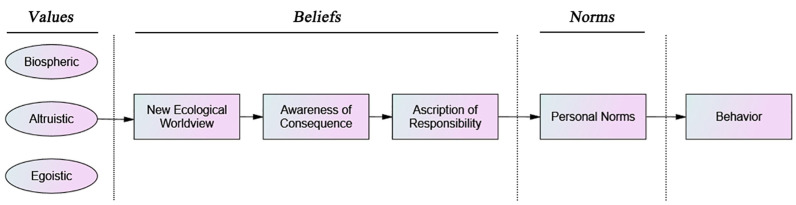
The Value-Belief-Norm Theoretical Model [29].

The VBN theory, which was first proposed by Stern et al. [26], explains how values affect behavior within an environmentalist context. This theory combines three theories, the Value System Theory of Schwartz [28], the New Ecological Paradigm (NEP) of Dunlap [50] and the Norm Activation Theory (NAT) of Schwartz [27]. According to the VBN theory, individual norms are a significant factor in the development of environmentally friendly behavior. The ecological worldview, awareness of consequence and ascription of responsibility are the three beliefs that contribute to the formation of norms, which are then further developed by three values [26,29,51]. The VBN theory includes a number of significant ideas (such as values and an ecological worldview) that are essential to environmentalism and is mainly geared to assess environmentally friendly actions. In light of this, the VBN theory proposes a chain of causality consisting of values, beliefs, norms and behaviors [26,29,52] (Figure 1). 

In the existing tourism literature, the VBN theory has been employed in various contexts to explain the pro-environmental behavior of museum vacationers [53], adventure tourists [54] and visitors to green accommodation [51], national parks [55] and conservation areas [56], sustainable transportation [57] and towel reuse intention [58]. In recent studies, Megeirhi et al. [59] applied the VBN theory to sustainable heritage tourism and Zinan Zhao et al. [60] examined agritourism consumer intentions by combining VBN and planned behavior theories. Due to environmental concerns and a sense of moral responsibility, tourists today tend to adopt a sustainable mindset. Nevertheless, few researchers have considered the VBN theory as an integrated approach to university students’ environmentally sustainable behavior toward tourism destinations. Hence, this study aims to create an integrated model that will improve university students’ understanding of environmentally sustainable behavior.

### 2.3. Environmentally Sustainable Behavior

The concept of sustainability has become universal and an integral part of many sectors’ policies. Environmental sustainability aims to satisfy the demands of current and future generations about resources and services without compromising ecosystems. Engaging in sustainable behavior, as the definition of sustainability indicates, is actually aimed at enhancing overall life satisfaction for individuals in the long term [61,62]. By adopting pro-environmental behavior patterns, individuals can make a significant contribution to long-term environmental sustainability [63]. However, on an individual level, coping with environmental sustainability is a hard issue. The environmental awareness and values of an individual are critical components of the environmental sustainability movement [52,64,65].

To better understand how values, beliefs and pro-environmental behaviors are related, several studies have been conducted [66,67]. Prior research has examined residents’ attitudes toward sustainable tourism [68], travel modes that are environmentally friendly [64], dedication to biodiversity and evaluation of environmental risk [67,69,70]. However, the environmentally sustainable behavior of university students is still very rarely studied. Therefore, further study is required to address the driving forces behind the behavior of university students to promote sustainable values toward tourism destinations [66,71]. This study suggests an integrated framework for a more in-depth assessment of university students’ environmentally sustainable behavior. 

### 2.4. Conceptual Model and Hypothesis Development

**Figure 2:**
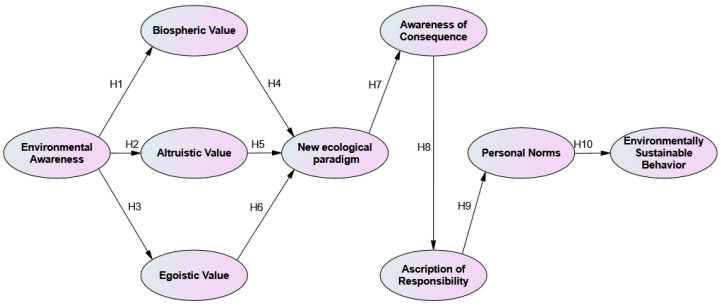
The conceptual model.

Using the VBN theory, this study examines university students’ environmentally sustainable behavior through the lens of their environmental awareness. Figure 2 illustrates the framework for the empirical analysis of this research.

#### 2.4.1. Relationship between Environmental Awareness and Values

Environmental awareness is a crucial component in improving environmental behavior. It does not directly influence environmental-friendly behavior but does so indirectly via other factors [15,72,73,74]. Based on previous studies, environmental awareness has an essential role in environmental values [42,75]. According to VBN theory, environmental values are crucial for triggering a person’s sense of moral obligation to protect the environment [26]. A person’s values also serve as a guide for understanding the world in a more comprehensive way. These three dimensions (biosphere, altruistic and egoistic) are important for guiding an individual’s behavior [15,26]. Biospheric value refers to the significance of living in harmony and interdependence with the natural environment. Altruistic value refers to selfless action in which the good of another is placed before one’s own interest. Egoistic value refers to the importance of self-interest over society’s interests [29]. In previous studies, individuals with environmental awareness that are influenced by biospheric value tend to take positive environmental action [32,76]. Environmental awareness and altruism are positively correlated in the context of green intentions [77,78,79]. A higher level of environmental awareness and higher levels of egoistic value are associated with tourists’ intentions to visit green hotels and positively influence tourists’ pro-environmental behaviors [55,80,81,82]. As a result, the following hypotheses are proposed:

**H1.**  *Environmental awareness positively influences the biospheric value of university students.*

**H2.**  *Environmental awareness positively influences the altruistic value of university students.*

**H3.**  *Environmental awareness positively influences the egoistic value of university students.*

#### 2.4.2. Relationship between Values and the NEP

Value systems strongly influences people’s beliefs. As Stern et al., pointed out [26], three values contribute to individuals’ beliefs about pro-environmental behavior. Several studies suggest that values are important in the development of the NEP [83,84]. The NEP is used to measure the general beliefs related to how people perceive nature-human relationships. The NEP is described as those who believe that humans can disrupt nature’s balance, that there are limitations to human society’s expansion and that humans possess a right to dominate the rest of the natural world [50].

It is stated that psychological behavior is influenced by people’s beliefs and biospheric values. According to Chua et al. [85], the NEP mediates both the relationship between biospheric value and personal norms, as well as egoistic value and personal norms. Tourism’s altruistic value has been shown to significantly affect the NEP [20,86]. From the viewpoint of tourism, earlier empirical research discovered substantial associations between egoistic value and the NEP [20,32,55,64,66,87]. Thus, this study proposes the following hypotheses:

**H4.**  *Biospheric value positively influences the NEP of university students.*

**H5.**  *Altruistic value positively influences the NEP of university students.*

**H6.**  *Egoistic value positively influences the NEP of university students.*

#### 2.4.3. Relationship between the NEP and Awareness of Consequence

Awareness of consequence refers to one’s awareness of the negative effects of antisocial behavior [29]. Based on VBN theory, awareness of consequence is a cognitive prerequisite for activating moral norms and is necessary for individuals to behave in an environmentally friendly way. Van Riper and Kyle [32] examined travelers’ eco-friendly behavior. They found that NEP positively influences awareness of consequence. Furthermore, in their research, Kiatkawsin and Han [66] discovered that NEP was responsible for 33.8% of the variance in awareness of consequence. Based on this, this study hypothesizes that:

**H7.**  *The NEP positively influences the awareness of consequence of university students.*

#### 2.4.4. Relationship between Awareness of Consequence and Ascription of Responsibility

The definition of ascription of responsibility is that human behavior can help to mitigate the impacts of environmental issues [26]. It has been found that awareness of consequence and ascription of responsibility are positively correlated in a variety of contexts related to pro-environmental behavior, such as museum vacationers [53], tourists’ pro-sustainable behavior [20,66], cruise travelers’ eco-friendly behavior [88] and adventure tourism [54]. Thus, the proposed hypothesis is as follows:

**H8.**  *Awareness of consequence positively influences the ascription of responsibility of university students.*

#### 2.4.5. Relationship between Ascription of Responsibility and Personal Norms

Based on VBN theory, three beliefs-NEP, awareness of consequence and ascription of responsibility have positive impacts on personal norms [26]. Holding an ascription of responsibility creates a sense of moral obligation and affects one’s personal norms [89]. Han [51] and Choi [76] found that personal norms are positively influenced by ascription of responsibility when it comes to green accommodation intentions. In addition, Han et al. [88], as well as Wensing et al. [90], also confirmed these findings. Accordingly, this study proposes the following hypothesis:

**H9.**  *Ascription of responsibility positively influences the personal norms of university students.*

#### 2.4.6. Relationship between Personal Norms and Environmentally Sustainable Behavior

In contrast to attitudes, moral obligation tends to remain relatively stable [91], hence offering a more suitable leverage point for behavior change. Prior studies have found that personal norms are associated with environmentally sustainable behavior, such as conserving water, avoiding littering, turning off lights and taking public transportation [14,86,92]. 

Researchers across various contexts have empirically validated this relationship [50,93]. Minton and Rose [94] found that people’s personal norms were instrumental in determining their green purchasing behavior. When choosing environmentally friendly travel models, personal norms have a crucial role in decision-making [83,95]. Based on this, the following hypothesis was proposed:

**H10.**  *Personal norms positively influence the environmentally sustainable behavior of university students.*

## 3. Materials and Methods

### 3.1. Participants

The participants were all university students at an eastern Chinese university with at least one travel experience. They were recruited through convenience sampling on campus. A total of 320 students took part in the research; eight invalid questionnaires and 11 questionnaires with zero trips were excluded and 301 valid replies were eventually utilized for data analysis. The final sample included 133 males (44.2%) and 168 females (55.8%). Among these 301 students, 21.9% (66) were freshmen, 15% (45) were sophomores, 40.9% (123) were juniors, 17.9% (54) were seniors and 4.3% (13) were graduate students (Table 1).

### 3.2. Measures

There are nine constructs in the study model, including environmental awareness, biospheric value, altruistic value, egoistic value, new ecological paradigm, awareness of consequence, ascription of responsibility, personal norms and environmentally sustainable behavior. All of the variables in the model’s VBN framework were reflective in nature. Some items were eliminated because they had a loading of less than 0.6, which was essential for the reflective measurement model to establish validity and reliability. For measuring model constructs, items were taken from previous studies. The environmental awareness (three items) was constructed based on Xu et al. [15], Panda et al. [77] and Ballantyne et al. [96]. The three items of biospheric value were referenced from Groot and Steg [52] and Stern et al. [26]. The altruistic value (three items) was constructed based on Stern et al. [26], Groot et al. [52], Riper et al. [32]. The three items of egoistic value were adapted from Stern et al. [26]. The NEP (three items) was developed from Dunlap et al. [30,50]. The three items of awareness of consequence were constructed based on Stern et al. [26] and Landon et al. [20]. Ascription of responsibility (three items) was constructed referring to Landon et al. [20] and Steg and Groot [97]. Personal norms (three norms) were derived from Landon et al. [20], Steg and Groot [97] and Wu et al. [98]. Environmentally sustainable behavior (three items) was referenced from Groot and Steg [52], Stern [29] and Paswan et al. [65]. 27 items were measured on a 5-point Likert scale, ranging from “1 = strongly disagree” to “5 = strongly agree”. 

A translation and back translation method was conducted to translate the survey questionnaire from English to Chinese. To ensure the measuring scales’ content validity, twenty graduate students with tourism majors underwent testing. To improve the clarity of the process, some unclear items have been reworded and modified slightly.

### 3.3. Data Analysis

The data analysis was conducted through a two-step procedure [99]. In the first step, a confirmatory factor analysis was conducted to evaluate the hypothesized factor structure for its overall model fit, construct reliability and construct validity. Structural equation modeling (SEM) was used in the second step to test the hypothesized structural relationships. SPSS 26.0 and AMOS 24.0 were used to analyze the collected data.

## 4. Results 

### 4.1. Measurement Model 

In the initial tests of the measuring model, discriminant validity was inadequate between the constructs ascribed responsibility and personal norms according to the established standards (e.g., both constructs had a higher squared correlation than the Average Variance Extracted (AVE)). Thus, the construct of ascription of responsibility has been eliminated. Despite theory-based hypotheses, post hoc model modifications are frequently needed in practice. Table 2 displays the findings of the general features of the structural variables. For internal consistency, all scales’ Cronbach’s alpha values were higher than the suggested cutoff value of 0.7. [100], varying from 0.750 to 0.832. Above the recommended minimum of 0.60, the constructions’ composite reliability (CR) ratings varied from 0.753 to 0.836. The factor loadings ranged from 0.602 to 0.891, above the recommended levels of 0.60 for established items [101,102]. Additionally, the AVE assessed the convergent validity of the constructs. The findings revealed that the computed AVE values, ranging from 0.505 to 0.633, were all more than 0.50. The correlation coefficients between the latent variables were smaller than the square root of AVE on the bold diagonal, as shown by the findings (see Table 3), supporting the discriminant validity [102].

### 4.2. Structural Model

A SEM analysis was performed to validate the extended VBN model and to test if causal relationships were established between the theoretical variables. The analysis showed that the reduced model suited the data well [103]: chi-square (x2) = 485.766; degrees of freedom (df) = 243; x2/df = 1.999; probability level (*p*) = 0.000; RMR = 0.034; GFI = 0.880; AGFI = 0.852; NFI = 0.846; TLI = 0.904; CFI = 0.915; RMSEA = 0.058. 

The resulting path coefficients showed how the variables were related following the hypothesis test. Table 4 shows the hypothesis results. First, environmental awareness was found to significantly predict (H1) biospheric value (β = 0.676; t = 7.699; *p* < 0.001), (H2) altruistic value (β = 0.611; t = 6.54; *p* < 0.001) and (H3) egoistic value (β = 0.233; t = 6.754; *p* < 0.001). Biospheric value was most affected by environmental awareness. Second, (H4) biospheric value affects NEP significantly (β = 0.599; t = 6.837; *p* < 0.001). Third, the NEP was found to predict (H7) awareness of consequence (β = 0.799; t = 8.240; *p* < 0.001). Forth, the effects of (H9) awareness of consequence on personal norms (β = 0.635; t = 7.454; *p* < 0.001) were statistically significant. Lastly, the hypothesized relationship between (H10) personal norms and environmentally sustainable behavior (β = 0.741; t = 9.222; *p* < 0.001) were confirmed. However, the effect of (H5) altruistic value (β = 0.114; t = 1.600; *p* > 0.05) and (H6) egoistic value (β = 0.104; t = 1.783; *p* > 0.05) to NEP were not significant. As ascription of responsibility was removed from the model, no evidence was found to confirm its hypothesized relationships with awareness of consequence or personal norms.

**Figure 3:**
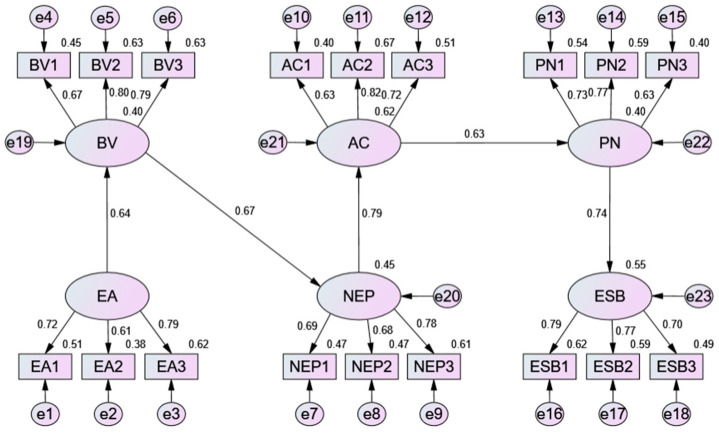
The SEM results of revised model.

Two insignificant pathways were removed from the original model. As well, the revised model fit the research data well: chi-square (x2) = 264.897; degrees of freedom (df) = 130; x2/df = 2.038; probability level (*p*) = 0.000; RMR = 0.028; GFI = 0.908; AGFI = 0.879; NFI = 0.883; TLI = 0.937; CFI = 0.936; RMSEA = 0.059. Therefore, the revised model was selected as the final model for further analysis. Figure 3 displays the SEM outcomes of the revised model.

Table 5 shows the final model’s variable path coefficients. Specifically, the impact of environmental awareness on biospheric value is β = 0.636 (t = 7.395, *p* < 0.001). The impact of biospheric value on NEP is β = 0.671 (t = 8.390, *p* < 0.001). The impact of NEP on awareness of consequence is β = 0.786 (t = 8.700, *p* < 0.001). The impact of awareness of consequence on personal norms is β = 0.632 (t = 6.974, *p* < 0.001). Lastly, the effect of personal norms on environmentally sustainable behavior is β = 0.741 (t = 7.931, *p* < 0.001). 

### 4.3. Indirect-Impact Assessment

The proposed model’s mediating relationships between variables were tested using bootstrapping analysis (Table 6). The findings demonstrated that the indirect effects of biospheric value, the NEP, awareness of consequence and personal norms were significant, as zero is not included in the 95% confidence interval for any indirect impact. Each indirect effect was also evaluated for its *p*-value, as shown in Table 5. The *p*-value of the indirect effect of environmental awareness on the NEP, biospheric value on awareness of consequence, the NEP on personal norms and awareness of consequence on environmentally sustainable behavior showed significant results. The study next evaluated the significance of the direct relationship among the relationships mentioned above. As shown in Table 6, although there were weak correlations between environmental awareness and the NEP, awareness of consequences and environmentally sustainable behavior, a statistically significant correlation was found between biospheric value and awareness of consequence, the NEP and personal norms. The results show that biospheric value fully mediates the relationship between environmental awareness and the NEP, personal norms fully mediates the relationship between awareness of consequence and environmentally sustainable behavior because indirect relationship is significant and direct relationship is insignificant, whereas the NEP partially mediates the relationship between biospheric value and awareness of consequence; awareness of consequence partially mediates the relationship between the NEP and personal norms since both the direct and indirect effects are significant.

## 5. Discussion

Based on their education, university students can make a valuable contribution to promoting environmental sustainability. Their behavior has been studied to some extent in previous studies of sustainable tourism in general level. In order to gain a deeper understanding, this study investigated university students’ environmentally sustainable behavior towards tourism destinations.

### 5.1. Theoretical Implications

This study’s theoretical achievement is the construction of a framework to explain the formation of Chinese university students’ environmentally sustainable behavior as tourists. This framework was developed by integrating VBN factors (i.e., biospheric value, altruistic value, egoistic value, the NEP, awareness of consequence, personal norms) with environmental awareness. The proposed model to an undergraduate population is suitable for predicting environmentally sustainable behavior. Empirical data provide strong support for the study’s findings. The following provides a more thorough explanation of the factors that affect university students’ participation in environmentally sustainable behavior.

#### 5.1.1. The Influence of Environmental Awareness on University Students’ Environmentally Sustainable Behavior

As a result of the study, environmental awareness positively impacted biospheric value, altruistic value and egoistic value (Table 4). The environmental values of tourism destinations were more important to university students with high environmental awareness. Consequently, environmental awareness changes the beliefs of university students in terms of their norms, behavior and self-efficacy. The results were supported by previous researches which found that individuals who were more aware of the environment cared more about eco-friendly products [15,68].

As shown in Table 2, the university students had a high degree of environmental awareness (mean = 4.29). Humans are intrinsically aware of the environment, but it may not have a direct impact on environmentally sustainable behavior. According to previous research [56], environmental awareness indirectly affects behavior through the VBN variables of values and beliefs, which have a mediating effect. Therefore, in order to motivate more university students to engage in environmentally sustainable behavior, it is crucial to develop public policies that increase environmental awareness. 

In general, the SEM results support environmental awareness as a prerequisite variable of the extended VBN model when predicting university students’ environmentally sustainable behavior toward tourism destinations.

#### 5.1.2. The Influence of VBN Factors on University Students’ Environmentally Sustainable Behavior

The findings of this study indicate a direct and positive relationship between VBN factors and university students’ engagement in environmentally sustainable behavior, which is consistent with previous research [55,104,105].

Firstly, only the biospheric value was mediated by the NEP. Individuals who place a high value on the biosphere are likely to form positive perceptions about the human–environment relationship and to be more concerned about the natural world. However, altruistic and egoistic values did not correlate with the NEP. It appears that university students’ altruistic value and egoistic value are not significantly related to their capacity to develop an ecological worldview. There have been a few studies based on VBN that have come to the same conclusion. Kiatkawasin et al. [66], Han et al. [88] and Nordfjærn et al. [57] failed to find a connection between egoistic value and the NEP. Stern et al. [26], Steg et al. [106] and Sahin et al. [107] concluded that altruistic value failed to explain the NEP. However, some studies have confirmed this relationship [55,56,81]. In-depth research is needed in future study. 

Secondly, the findings showed that the indirect effects of biospheric value, the NEP, awareness of consequence and personal norms were significant. Based on the finding, it was concluded that university students having high biospheric value, environmental worldview and awareness of problems are likely to be morally responsible for acting in a way that is environmentally sustainable. Specially, when people are faced with environmental issues, the NEP serves as a filter between their values and norms. Previous studies have also reported similar findings [83,108,109,110]. 

Thirdly, it was established that ascription of responsibility and personal norms lack discriminant validity. The finding was supported by some studies. Van Riper and Kyle [32] identified that ascribed responsibility accounted for 82% of the variance in personal norms. According to Raymond et al. [111] and Landon et al. [112], past studies using the Norm Activation Theory have found that ascribed responsibility and personal norms have very strong correlations [97]. It will be necessary to verify the measurement properties of both scales in future VBN applications. 

Lastly, the examination on the details of environmentally sustainable behavior found interesting findings. Low loadings caused two items to be removed from the research. They were (1) I would purchase environmentally friendly tourism products regardless of price and (2) I would prefer to purchase locally produced crafts and goods. Actions associated with potential price increases resulted in less than satisfactory loadings [113]. The results are consistent with Kollmuss and Agyeman’s conclusion that a shortage of money hinders environmentally sustainable behavior, regardless of attitudes. These results are affected by economic barriers since the study group was young university students.

### 5.2. Practical Implications

It is important for universities and environmentally relevant departments to promote environmentally sustainable behaviors among university students based on the theoretical findings. 

As environmental awareness contributes positively to the development of environmentally sustainable behavior among university students, universities should include sufficient environment courses as compulsory part of their programs to help students raising their awareness of the environment and sense of responsibility. Given the results conducted on this study, students’ environmentally sustainable behavior can be fostered indirectly by enhancing students’ biospheric value. The curriculum at universities should have a stronger connection to environmental welfare. Environmental awareness and environmental responsibility should be fostered in students in order to help them deal with the current environmental crisis. Universities should collaborate more closely with local authorities, communities and non-governmental organizations (NGOs) to provide students with various education forms, for instance, community-based [114] and project-based [115]. These forms can help students learn how environmental problems negatively affect nature, society, communities and individuals, as well as establish personal norms for acting in environmentally sustainable behavior. 

Sustainability should also be incorporated into tourism curricula and courses. The practice of sustainability not only advances society but also enhances and safeguards students’ personal lives. There are a few empirical studies that support this claim. The consumption of environmentally friendly products was associated with greater personal well-being [116], higher satisfaction with overall life [117] and greater happiness [118]. Universities may play a key role in creating both individual and societal learning systems for sustainable development thanks to academic freedom and autonomy [119]. 

Several studies have indicated that mass media influence students’ academic performance [120]; thus, mass media should be used more intensively to transmit environmental information to promote students’ environmentally sustainable behavior. However, mass media should provide society with accurate, timely information. Various approaches should be taken from an administrative and educational standpoint to disseminate environmental awareness information, including audio, images, animations, videos and interactive content. Further, departments should also listen to student opinions [63] so that students will feel more confident in solving environmental problems and establish personal norms toward environmental protection.

## 6. Conclusions

Using the VBN theory, this study explores university students’ environmentally sustainable behavior through the influencing factor of environmental awareness. The results exhibited the positive direct relationship between environmental awareness and biosphere, altruistic and egoistic values. The research also found that environmental awareness, biosphere values, beliefs and personal norms are highly predictive of environmentally sustainable behavior. For this reason, initiatives promoting environmentally sustainable behavior among university students must take environmental awareness into account. The extended model would be a guide for developing mainstream environmental education and disseminating information through mass media on environmental awareness for university students.

Even though this study yielded positive results, it does have some limitations. Despite being statistically adequate, the study has limited generalizability due to its relatively small sample size. Future sample size expansion will be required to include a sizable number of university students from various Chinese universities in order to draw more firm findings. Second, this study only examined university students’ environmental sustainability behavior by taking into account one external factor (environmental awareness); further studies may include other factors, such as environmental sensitivity, environmental ethics. Third, a common problem with survey-based research is the lack of observational behavior and reliance on self-reporting alone. This means that the results can be subjectively biased. Future research should combine survey-based methods with other methods (such as participant observation) and adopt more mixed methods to correct for the bias in results from survey-based research methods. 

## Figures and Tables

**Table 1:** Summary of the demographic statistics (*n* = 301).

Characteristics	Category	Frequency	Percentage (%)
Gender	male students	133	44.2
	female students	168	55.8
Grade	freshmen	66	21.9
	sophomores	45	15.0
	juniors	123	40.9
	seniors	54	17.9
	graduate students	13	4.3
Major	humanities	162	53.8
	science	70	23.3
	engineering	69	22.9

**Table 2:** Summary of the demographic statistics (*n* = 301).

Construct	Items	Mean	S.D.	t-Value	S.F.L.	C.R.	AVE	Cronbach’s Alpha (α)
EA	EA1	4.33	0.58		0.764	0.755	0.507	0.751
	EA2	4.00	0.65	8.627	0.649			
	EA3	4.53	0.59	8.765	0.719			
BV	BV1	4.56	0.55		0.632	0.801	0.576	0.795
	BV2	4.63	0.53	9.889	0.834			
	BV3	4.62	0.55	10.003	0.796			
AV	AV1	4.41	0.58		0.628	0.753	0.505	0.750
	AV2	4.40	0.61	8.451	0.770			
	AV3	4.28	0.62	8.562	0.727			
EV	EV1	3.07	0.88		0.891	0.836	0.633	0.832
	EV2	2.81	0.95	12.898	0.786			
	EV3	3.53	0.83	11.875	0.697			
NEP	NEP1	4.60	0.56		0.602	0.777	0.541	0.771
	NEP2	4.41	0.62	9.111	0.745			
	NEP3	4.48	0.62	8.760	0.841			
AC	AC1	4.16	0.62		0.676	0.780	0.545	0.773
	AC2	4.41	0.61	9.216	0.863			
	AC3	4.46	0.61	9.464	0.659			
PN	PN1	4.13	0.70		0.748	0.764	0.520	0.760
	PN2	4.14	0.67	9.113	0.764			
	PN3	4.17	0.72	8.934	0.645			
ESB	ESB1	4.17	0.68		0.727	0.798	0.569	0.797
	ESB2	4.15	0.67	10.524	0.805			
	ESB3	4.35	0.67	10.453	0.728			

Note: EA = environmental awareness, BV = biospheric values, AV = altruistic value, EV = egoistic value, NEP = new ecological paradigm, AC = awareness of consequence, PN = personal norms, ESB = environmentally sustainable behavior.

**Table 3:** Discriminant validity.

	AVE	ESB	PN	AC	NEP	EV	AV	BV	EA
ESB	0.569	**0.754**							
PN	0.520	0.714	**0.721**						
AC	0.545	0.490	0.563	**0.738**					
NEP	0.541	0.501	0.547	0.709	**0.736**				
EV	0.633	0.194	0.220	0.214	0.126	**0.796**			
AV	0.505	0.334	0.345	0.473	0.404	0.190	**0.711**		
BV	0.576	0.435	0.442	0.574	0.613	0.038	0.643	**0.759**	
EA	0.507	0.282	0.326	0.569	0.410	0.256	0.533	0.625	**0.712**

Note: ESB = environmentally sustainable behavior, PN = personal norms, AC = awareness of consequence, NEP = new ecological paradigm, EV = egoistic value, AV = altruistic value, BV = biospheric values, EA = environmental awareness, diagonals (in bold) represent the square root of the AVE.

**Table 4:** Standardized path coefficients and hypothesis testing results.

Path	Unstandardized Coefficient (B)	Standardized Coefficient (β)	S.E.	t	Hypothesis
EA → BV	0.629	0.676 ***	0.082	7.699	H1: Supported
EA → AV	0.565	0.611 ***	0.084	6.754	H2: Supported
EA → EV	0.463	0.233 ***	0.138	3.345	H3: Supported
BV → NEP	0.62	0.599 ***	0.091	6.837	H4: Supported
AV → NEP	0.118	0.114	0.074	1.600	H5: Not supported
EV → NEP	0.051	0.104	0.028	1.783	H6: Not supported
NEP → AC	0.81	0.799 ***	0.098	8.240	H7: Supported
AC → PN	0.84	0.635 ***	0.113	7.454	H9_rev_: Supported
PN → ESB	0.766	0.741 ***	0.083	9.222	H10: Supported

Note: EA = environmental awareness, BV = biospheric values, AV = altruistic value, EV = egoistic value, NEP = new ecological paradigm, AC = awareness of consequence, PN = personal norms, ESB = environmentally sustainable behavior, H8 was eliminated because of the removal of ascription of responsibility, H9 is revised hypothesis, *** = *p* < 0.001.

**Table 5:** The estimate of path coefficients for the revised model.

Path	Unstandardized Coefficien (B)	Standardized Coefficient (β)	S.E.	t
EA → BV	0.566	0.636 ***	0.077	7.395
BV → NEP	0.889	0.671 ***	0.106	8.390
NEP → AC	0.627	0.786 ***	0.072	8.700
AC → PN	0.740	0.632 ***	0.106	6.974
PN → ESB	0.762	0.741 ***	0.096	7.931

Note: EA = environmental awareness, BV = biospheric values, NEP = new ecological paradigm, AC = awareness of consequence, PN = personal norms, ESB = environmentally sustainable behavior, *** = *p* < 0.001.

**Table 6:** Results of indirect effect.

Relationship	Direct Effect	95% Confidence Interval	Significance (*p* < 0.05)	Indirect Effect	95% Confidence Interval	Significance (*p* < 0.05)
EA→NEP	0.113	−0.217, 0.397	No	0.430	0.263, 0.716	Yes
BV→AC	0.293	0.056, 0.555	Yes	0.327	0.188, 0.533	Yes
NEP→PN	0.298	0.022, 0.576	Yes	0.169	0.054, 0.380	Yes
AC→ESB	0.179	−0.055, 0.431	No	0.255	0.069, 0.535	Yes

Note: EA = environmental awareness, BV = biospheric values, NEP = new ecological paradigm, AC = awareness of consequence, PN = personal norms, ESB = environmentally sustainable behavior.

## Data Availability

Not applicable.
